# Rapid identification of nine species of diphyllobothriidean tapeworms by pyrosequencing

**DOI:** 10.1038/srep37228

**Published:** 2016-11-17

**Authors:** Tongjit Thanchomnang, Chairat Tantrawatpan, Pewpan M. Intapan, Oranuch Sanpool, Viraphong Lulitanond, Somjintana Tourtip, Hiroshi Yamasaki, Wanchai Maleewong

**Affiliations:** 1 Faculty of Medicine, Mahasarakham University, Mahasarakham, 44000, Thailand; 2 Research and Diagnostic Center for Emerging Infectious Diseases, Khon Kaen University, Khon Kaen, 40002, Thailand; 3 Division of Cell Biology, Department of Preclinical Sciences, Faculty of Medicine, Thammasat University, Rangsit Campus, Pathum Thani, 12120, Thailand; 4 Department of Parasitology, Faculty of Medicine, Khon Kaen University, Khon Kaen, 40002, Thailand; 5 Department of Microbiology, Faculty of Medicine, Khon Kaen University, Khon Kaen, 40002, Thailand; 6 Department of Parasitology, National Institute of Infectious Diseases, Tokyo, 162-8640, Japan

## Abstract

The identification of diphyllobothriidean tapeworms (Cestoda: Diphyllobothriidea) that infect humans and intermediate/paratenic hosts is extremely difficult due to their morphological similarities, particularly in the case of *Diphyllobothrium* and *Spirometra* species. A pyrosequencing method for the molecular identification of pathogenic agents has recently been developed, but as of yet there have been no reports of pyrosequencing approaches that are able to discriminate among diphyllobothriidean species. This study, therefore, set out to establish a pyrosequencing method for differentiating among nine diphyllobothriidean species, *Diphyllobothrium dendriticum, Diphyllobothrium ditremum, Diphyllobothrium latum, Diphyllobothrium nihonkaiense, Diphyllobothrium stemmacephalum, Diplogonoporus balaenopterae, Adenocephalus pacificus*, *Spirometra decipiens* and *Sparganum proliferum*, based on the mitochondrial cytochrome *c* oxidase subunit 1 (*cox1*) gene as a molecular marker. A region of 41 nucleotides in the *cox1* gene served as a target, and variations in this region were used for identification using PCR plus pyrosequencing. This region contains nucleotide variations at 12 positions, which is enough for the identification of the selected nine species of diphyllobothriidean tapeworms. This method was found to be a reliable tool not only for species identification of diphyllobothriids, but also for epidemiological studies of cestodiasis caused by diphyllobothriidean tapeworms at public health units in endemic areas.

The order Diphyllobothriidea (Platyhelminthes: Cestoda) is a large group of tapeworms that parasitize mammals, birds, amphibians and reptiles^1^,^2^. The order also comprises several important genera of human parasites, such as *Diphyllobothrium*, *Adenocephalus*, *Diplogonoporus* and *Spirometra*^3^,^4^,^5^,^6^. Diphyllobothriasis is a zoonosis of which adult-stage tapeworms of the genus *Diphyllobothrium*, specifically *Diphyllobothrium latum* and *D. nihonkaiense*, are the most frequent etiologic agents in humans^3^,^4^,^7^,^8^. *Diphyllobothrium dendriticum*^6^, *Adenocephalus pacificus* (syn. *Diphyllobothrium pacificum*^6^), and *Diphyllobothrium stemmacephalum*^9^ have rarely been detected in humans. The life cycle of the *Diphyllobothrium* tapeworm consists of intermediate hosts (copepods and fish) and definitive hosts (e.g., marine mammals, carnivores, humans and piscivorous birds). Once the eggs hatch in water, the ciliated coracidia are ingested by copepods and develop into procercoid larvae. When infected copepods are engulfed by a second intermediate host, the procercoid larva migrates to the musculature and the body cavity (mainly stomach, liver and other organs) and develops into a plerocercoid larva^4^. Humans become infected by eating raw or undercooked fish containing plerocercoids, which subsequently develop into adult tapeworms in the human intestine^4^. Symptoms of diphyllobothriasis include diarrhoea, loose bowels and light abdominal pain or discomfort. Most of the cases are asymptomatic. Heavy infections may cause intestinal obstruction, and motile proglottids can result in cholecystitis or cholangitis. Prolonged or severe *D. latum* infection can cause megaloblastic anaemia^4^,^6^.

Human diplogonoporiasis refers to a tapeworm infection caused by *Diplogonoporus balaenopterae* (syn. *Diplogonoporus grandis*^10^), which usually infects minke whale, sei whale and hump back whale^11^ and has been found almost exclusively in Japan^10^,^12^,^13^. The details of its life cycle are not clear, but various marine fish, such as raw whitebait, are suspected as potential sources of human infection^14^,^15^. Human sparganosis is a harmful infective zoonosis caused by plerocercoid larvae (e.g., sparganum) belonging to the genus *Spirometra* or *Sparganum*. Sparganosis is classified into two forms, nonproliferative and proliferative. The former is caused by *Spirometra erinaceieuropaei* and *Spirometra decipiens* in China^16^, Japan^17^, Korea^18^,^19^, Taiwan^20^ and Thailand^21^ and *Spirometra mansonoides* in the USA^22^. The latter is caused by the more pathogenic and disseminating *Sparganum proliferum*, which is reported as occurring only sporadically in Japan^23^, Taiwan^24^ and Thailand^25^ and very rarely in Paraguay, Venezuela, and the USA^26^,^27^. *Spirometra* species require two intermediate hosts for the completion of their life cycle. Copepods serve as the first intermediate host for the procercoid larvae. Vertebrates (reptiles, amphibians, birds, and mammals) serve as the second intermediate and paratenic hosts for the plerocercoid larvae. Human infection occurs through the ingestion of water polluted with copepods carrying procercoids or through ingesting raw or undercooked meat such as frog, snake or chicken infected with plerocercoids^21^,^28^. The plerocercoid migrates predominantly to subcutaneous tissue, but it also affects the brain and eyes. Sparganum of *Spirometra* species can rarely develop into adult worms in the human intestine^29^. The details of the *Sp. proliferum* life cycle are not clear and its adult stage is unknown.

Since the eggs, larvae and adult tapeworms of the genera *Diphyllobothrium*, *Adenocephalus* and *Spirometra* are morphologically similar, their identification in human tissues is exceedingly difficult and requires special expertise. Only one study has successfully used morphometric and ultrastructural (surface morphology) egg features to distinguish among the 8 species of diphyllobothriids^30^. For these reasons, many molecular methods using different genetic markers have been developed for the identification of diphyllobothriid tapeworms: *cox1* for *Diphyllobothrium*^31^,^32^,^33^,^34^ and *S. erinaceieuropaei*^16^,^35^, *sdhB* for *Sp. proliferum* and *S. erinaceieuropaei*^36^, and *nad3* for *Sp. proliferum*^5^. These molecular methods are reliable and valuable tools for the identification of diphyllobothriidean tapeworms. Pyrosequencing is a DNA sequencing method that utilizes enzyme-coupled reactions and bioluminescence to monitor the pyrophosphate (PPi) release accompanying nucleotide incorporation using the synthesis of short nucleotide fragments that are directed by the sequence in real-time^37^. The method is principled on the real-time monitoring of 4 enzymes during DNA synthesis by luminescence using a step that results in a detectable light signal upon nucleotide incorporation. The detection is based on the PPi released when a nucleotide is incorporated into the DNA strand. The signal can be measured according to the number of bases added. This method can be used for mutation detection and single-nucleotide polymorphism (SNP) genotyping of large samples of screening material and high-throughput DNA analysis techniques^38^. The method, an alternative method, has been used for species-specific discrimination of *Entamoeba* species^39^ and for identification of various parasite taxa such as *Plasmodium*^40^, *Trichinella* species^41^, *Paragonimus*^42^ and lymphatic filaria^43^. Here we developed a pyrosequencing methodology that characterizes *cox1* gene amplicons for the identification of diphyllobothriidean tapeworms in humans and intermediate hosts.

## Results

**Figure 1:**
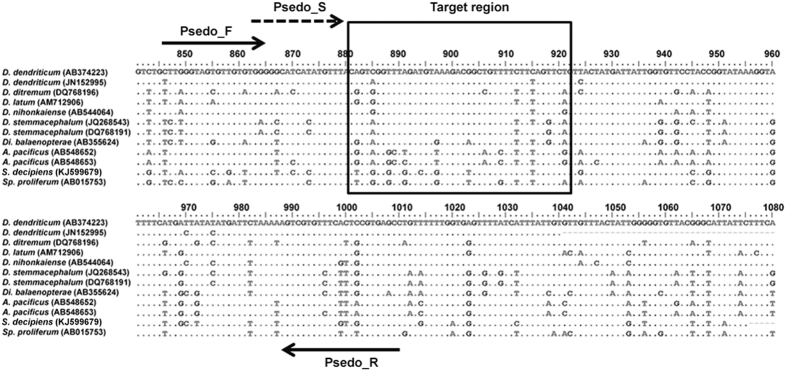
Multiple sequence alignments for indicating primer annealing positions and diagnostic target region. Nucleotide alignment of the mitochondrial *cox1* gene derived from *D. dendriticum, D. ditremum, D. latum*, *D. nihonkaiense*, *D. stemmacephalum*, *Di. balaenopterae*, *A. pacificus*, *S. decipiens* and *Sp. proliferum*. The solid arrows indicate the position of Psedo_F (forward primer) and biotinylated Psedo_R (reverse primer). The broken arrow indicates Psedo_S (sequencing primer). The boxed region shows the position (881–921) of the target area used for species-level identification. Dots indicate identical nucleotides between sequences. AM712906 (*D. latum*) is the reference sequence for nucleotides positions.

**Figure 2:**
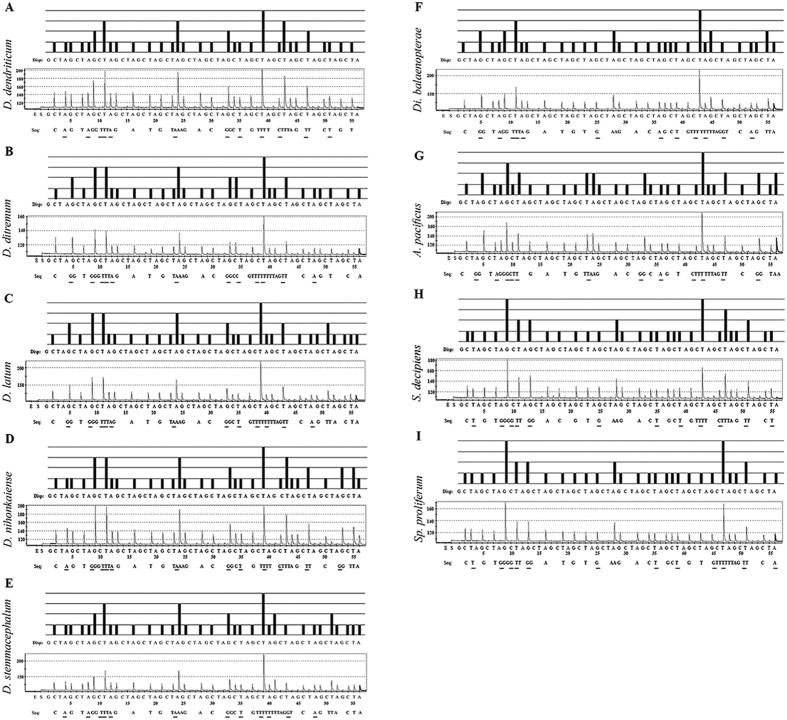
Theoretical pyrograms and pyrograms of control plasmids or analytical sample raw reads of 9 diphyllobothriid species. Pyrograms of the 41-base fragment of the *cox1* gene from *D. dendriticum* (**A**) *D. ditremum* (**B**) *D. latum* (**C**) *D. nihonkaiense* (**D**) *D. stemmacephalum* (**E**) *Di. balaenopterae* (**F**) *A. pacificus* (**G**) *S. decipiens* (**H**) and *Sp. proliferum* (**I**). Theoretical pyrogram patterns (top of each panel) and representative raw data of control plasmids or analytical samples from pyrosequencing (bottom of each panel) are shown. The actual sequence obtained by pyrosequencing is displayed below the panels following “Seq”. The Y-axis shows the level of fluorescence emitted by the incorporation of a nucleotide base, and the X-axis shows total number of bases added at that point in time; G, C, T, A, nucleotide bases. The underlined letters indicate the nucleotides used for identification of diphyllobothriidean tapeworms.

Based on sequence alignments of the *cox1* gene from the nine diphyllobothriid species shown in Table 1, the 41 nucleotides following the 3′ end of the sequencing primer presented high accuracy and were used as the target region for species identification (Fig. 1 and Table 2). The nucleotide sequence pattern in the target region of each diphyllobothriidean species is shown as pyrogram results (Fig. 2). Of the 41 nucleotides, 12 positions were sufficiently variable to discriminate among the diphyllobothriidean species (Tables 2 and 3). *Diphyllobothrium dendriticum* and *A. pacificus* were found to show intra-specific variation (Table 2). The pyrosequencing reproducibility was confirmed for the amplicons by Sanger sequencing conducted at First BASE Laboratories Sdn Bhd (Selangor, Malaysia) using the BigDye terminator v3.1 cycle-sequencing kit (Applied Biosystems (ABI), Carlsbad, CA), and both strands were directly sequenced using the PCR primers as sequencing primers (Model 310 or 3100, ABI), which yielded identical sequence data. No PCR products could be obtained from DNA samples of other parasites, human leukocytes or uninfected fish, proving the high specificity of the PCR primers.

## Discussion

The taxonomy of the diphyllobothriidean tapeworms generally depends on morphological classification, but morphological characteristics are not always decisive criteria, particularly in larval and immature worms, or inadequately preserved specimens. *Diphyllobothrium dendriticum*, *D. latum*, *D. nihonkaiense*, *A. pacificus* and *Spirometra* species are of medical importance and have caused an emerging public health problem, especially in countries in which fish consumption is expanding and new cooking habits involving raw or uncooked processing are gaining ground^6^,^33^,^44^. For the differential detection of diphyllobothriidean tapeworms in diagnostic laboratories, rapid, specific and cost-effective tools for routine diagnosis of parasites have been developed^6^,^8^,^33^. Molecular methods for differentiation among diphyllobothriideans are important in situations in which the morphology and epidemiological information are similar^6^,^34^,^45^,^46^. Many molecular methods have been developed for the identification of diphyllobothriidean tapeworms, for example, multiplex PCR for *D. latum*, *D. dendriticum*, *D. nihonkaiense*, and *A. pacificus*^33^, PCR for study of the taxonomic relationship between *Di. balaenopterae* and *Di. grandis*^10^, and PCR-restriction fragment length polymorphism for *Diphyllobothrium* and *Diplogonoporus* species^8^. However, the limitation with these methods is that analyzing the results requires gel electrophoresis, which is imprecise, has restricted throughput and can only be used to differentiate up to eight species^30^. Moreover, the differentiation among *D. ditremum, Di. balaenopterae*, *S. decipiens* and *Sp. proliferum* has not been evaluated. This prompted us to seek out a new tool for elucidation, namely pyrosequencing^47^.

The pyrosequencing operating system is divided into two modes, sequence analysis (SQA) and SNP. These are obtainable for handling, and each mode can detect sequenced nucleotides in a different manner. The SQA mode sequences short and medium- length DNA fragments (approximate size of 25–50 bp)^48^, whereas the SNP mode searches only for single base variations. Pyrosequencing has been used in various applications such as differentiation of *Rickettsia* species in hard tick samples^49^ and detection of benzimidazole-resistance- associated β-tubulin SNPs in cattle nematodes^50^. It is also suitable for identifying, screening and/or predicting resistant strains of bacteria for antibiotic drugs^51^. Here, we report on a new tool for rapid identification of nine diphyllobothriidean tapeworms using pyrosequencing. This method can identify *D. dendriticum*, *D. ditremum, D. latum*, *D. nihonkaiense*, *D. stemmacephalum, Di. balaenopterae*, *A. pacificus*, *S. decipiens* and *Sp. proliferum* (Table 1) at the species level. As another advantage of pyrosequencing, short PCR products are sufficient for this analysis. Thus, DNA from formalin-fixed samples that has degraded due to formalin fixation is suitable for pyrosequencing. In this study, we found that pyrosequencing was able to identify formalin-fixed *A. pacificus* (Fig. 2G).

The pyrosequencing technique developed in this study is a high-throughput tool that can be completed within 4 hours (excluding the DNA extraction period) and can achieve results from 96 samples simultaneously. The approximate estimated cost is $5 per test. However, the cost can vary depending upon the commercial origin of there agents used. In addition to its medical applications, this pyrosequencing technique can be useful for identification of larvae isolated from copepods, eggs, and adults among different taxa that share similar morphology. Pyrosequencing is a promising alternative high-throughput method for molecular genotyping of nine species of diphyllobothriidean tapeworms and can have important implications for epidemiological studies. This not only applies to endemic countries but also non-endemic areas in which travellers coming from endemic areas bring in the parasites^5^,^52^.

## Materials and Methods

### Parasite materials

Specimens of diphyllobothriidean tapeworms (larval and adult stages) were collected from intermediate and definitive hosts from different geographical localities (Table 1). A total of 29 diphyllobothriidean tapeworm specimens, including *D. dendriticum*, *D. ditremum, D. latum, D. nihonkaiense, D. stemmacephalum*, *Di. balaenopterae*, *A. pacificus*, *S. decipiens* and *Sp. proliferum* were examined (Table 1). The diphyllobothriid specimens examined in this study were fixed in 70% ethanol, with the exception of *A. pacificus,* which was fixed in 10% formalin.

Genomic DNA samples from non-diphyllobothriid parasites were used for the evaluation of primer specificity. These included samples from trematodes (*Opisthorchis viverrini*, *Clonorchis sinensis*, *Fasciola gigantica*, *Paragonimus heterotremus*, *Schistosoma japonicum*, *Schistosoma mekongi*, *Haplorchis taichui*, *Stellantchasmus* sp., *Centrocestus* sp., *Echinostoma malayanum*, intestinal lecithodendriid flukes), nematodes (*Trichuris trichiura*, hookworms, *Strongyloides stercoralis*, *Ascaris lumbricoides*, *Capillaria philippinensis*, *Trichostrongylus* sp.) and protozoa (*Isospora belli, Giardia duodenalis*). DNA samples from uninfected fish and human leukocytes were also analyzed.

### Ethical Approval

All methods in the study protocol were approved and were performed in accordance with the relevant guidelines and regulations of the ethical approval in the Ethics Committee in the National Institute of Infectious Diseases, Tokyo, Japan (No. 177). No experiment involving human subjects was used. All parasites were derived from left over specimens (informed consent was obtained from all subjects and the data were analyzed anonymously) or from specimens bought at markets. We confirmed that the process did not involve endangered or protected species.

### Primer design

Primer design was based on nucleotide alignment of *cox1* from *D. dendriticum*, *D. ditremum*, *D. latum*, *D. nihonkaiense*, *D. stemmacephalum*, *Di. balaenopterae*, *A. pacificus* (syn. *D. pacificum*); and *S. decipiens*, and *Sp. proliferum* (see Table 2 for GenBank accession nos.) The following primers were designed from *A. pacificus* sequence (GenBank accession no. AB548654): our primer sets (Psedo_F; 5′-TTTGGGTAGTGTTGTGTGGG-3′, corresponding to positions 846–865 and biotinylated Psedo_R; biotin-5′-GGCTCACGTAAAGAAACACGACT-3′, corresponding to positions 1010-988) and Psedo_S sequencing primer (5′-GGGGTCATCATATGTTTA-3′, corresponding to positions 863–880) were designed using PyroMark Q96ID software version 2.0 (Biotage, Uppsala, Sweden) (Fig. 1). *Diphyllobothrium latum* (GenBank accession no. AM712906) is the reference sequence that was used to determine the nucleotide positions.

### DNA extraction, plasmid preparation, DNA amplification by polymerase chain reaction (PCR) and pyrosequencing

Genomic DNA samples from 29 diphyllobothriid specimens (Table 1) were extracted using a DNeasy Blood & Tissue kit (Qiagen, Hilden, Germany). In addition, positive control plasmids for all diphyllobothriidean species were produced by ligation of each species-specific amplicon into a pGEM-T easy vector (Promega, WI), as has been described previously in other reports^53^. Each inserted gene was Sanger sequenced in both directions and the resulting sequences were identical to the gene sequences from which the primers were designed.

The *cox1* gene fragments (165 bp) were PCR amplified from the diphyllobothriidean DNA. The total PCR reaction volume was 25 μl and included 2 μl of the DNA template, 2.5 μl of 10× PCR buffer (Roche Applied Science, Mannheim, Germany), 0.2 mM of dNTP, 2 mM MgCl_2_, 0.2 μM of Psedo_F primer, 0.2 μM of biotinylated Psedo_R primer and 0.625 U of FastStart High Fidelity Enzyme Blend (Roche Applied Science). PCR was performed at 94 °C for 5 min followed by 35 cycles of denaturation at 95 °C for 30 sec, annealing at 48 °C for 30 sec, and extension at 72 °C for 30 sec, followed by a final extension at 72 °C for 10 min. PCR products were visualized using 1.5% agarose gel electrophoresis. After PCR amplification, biotinylated PCR products were added into 96-well plates and processed as described elsewhere^40^,^42^. The pyrosequencing system included DNA polymerase I, ATP sulfurylase, luciferase and apyrase. Pyrosequencing is a DNA sequencing technique that is based on the detection of released PPi during DNA synthesis. In a cascade of enzymatic reactions, visible light is generated that is proportional to the number of incorporated nucleotides. The cascade starts with a nucleic acid polymerization reaction in which inorganic PPi is released as a result of nucleotide incorporation by polymerase. The released PPi is subsequently converted to ATP by ATP sulfurylase, which provides the energy to luciferase to oxidize luciferin and generate light. Because the added nucleotide is known, the sequence of the template can be determined. Light is only generated when a newly added nucleotide is complementary to the next unpaired base in the template strand. The intensity of light is proportional to the number of sequential identical bases in a homopolymer, but determining the exact length of a homopolymer can be a problem for this technique^37^,^53^. In cases which the target sequence showed up to four homopolymers, the readout was analyzed manually to ensure accuracy.

## Additional Information

**How to cite this article**: Thanchomnang, T. *et al.* Rapid identification of nine species of diphyllobothriidean tapeworms by pyrosequencing. *Sci. Rep.* **6**, 37228; doi: 10.1038/srep37228 (2016).

**Publisher’s note**: Springer Nature remains neutral with regard to jurisdictional claims in published maps and institutional affiliations.

## Figures and Tables

**Table 1:** Diphyllobothriid samples tested by pyrosequencing.

Species	Stage	Host	Locality	Year of isolation
*D. dendriticum*	Plerocercoid	Glaucous gull (*Larus hyperboreus*)	Kansas, USA	2001
	Plerocercoid	Rainbow trout (*Oncorhynchus mykiss*)	Kelly Peterson Lake, Alaska, USA	2001
	Plerocercoid	*O. mykiss*	Lake Llanquihue, Los Lagos, Chile	2009
	Plerocercoid	*O. mykiss*	Lake Llanquihue, Los Lagos, Chile	2009
	Plerocercoid	*O. mykiss*	Lake Llanquihue, Los Lagos, Chile	2009
	Plerocercoid	*O. mykiss*	Lake Llanquihue, Los Lagos, Chile	2009
	Plerocercoid	*O. mykiss*	Lake Llanquihue, Los Lagos, Chile	2009
	Plerocercoid	*O. mykiss*	Lake Panguipulli, Los Rios, Chile	2014
*D. ditremum*	Plerocercoid	White char (*Salvelinus albus*)	Azabachye Lake, Kamchatka, Russia	1992
*D. latum*	Adult	Human	Santiago, Chile	2009
	Plerocercoid	Coho salmon (*Oncorhynchus kisutch*)	Lake Llanquihue, Los Lagos, Chile	2009
	Plerocercoid	*O. kisutch*	Lake Llanquihue, Los Lagos, Chile	2009
*D. nihonkaiense*	Adult	Human	Tokyo, Japan	2008
	Adult	Human	Tokyo, Japan	2008
	Adult	Human	Tokyo, Japan	2008
	Adult	Human	Tochigi, Japan	2008
	Adult	Human	Hamamatsu, Japan	2008
	Adult	Human	Tokyo, Japan	2009
	Adult	Human	Hamamatsu, Japan	2010
	Adult	Human	Nagasaki, Japan	2010
*D. stemmacephalum*	Adult	Human	Kanagawa, Japan	2015
*Di. balaenopterae*	Adult	Minke whale (*Balaenoptera acutorostrata*)	North Pacific Ocean	1997
	Adult	*B. acutorostrata*	North Pacific Ocean	1997
	Adult	*B. acutorostrata*	North Pacific Ocean	1997
*A. pacificus*	Adult	Human	Santiago, Chile	2000
*S. decipiens*	Plerocercoid	Japanese striped snake (*Elaphe quadrivirgata*)	Tokyo, Japan	2008
	Plerocercoid	*E. quadrivirgata*	Tokyo, Japan	2012
	Plerocercoid	*E. quadrivirgata*	Tokyo, Japan	2012
*Sp. proliferum*	Plerocercoid	Mouse (*Mus musculus*)	Origin is from Venezuela, maintained in Japan	2011

**Table 2:** Variable *cox1* nucleotide positions used for the differentiation of diphyllobothriidean tapeworms.

Species (Accession No.)	Nucleotide positions											
	882	885	888	889	891	897	903	906	909	912	918	921
*Diphyllobothrium dendriticum* (JN152995, AB374223, KC812048, AM412738)	A	A/C	T	T	A	A	G	T	T	C	T	T
*Diphyllobothrium ditremum* (DQ768196, FM209182)	G	G	T	T	A	A	G	C	T	T	T	A
*Diphyllobothrium latum* (AM712906*, AB269325)	G	G	T	T	A	A	G	T	T	T	T	A
*Diphyllobothrium nihonkaiense* (AB544064, AM412559)	A	G	T	T	A	A	G	T	T	C	T	G
*Diphyllobothrium stemmacephalum* (JQ268543, DQ768191)	A	A	T	T	A	A	G	T	T	T	G	A
*Diplogonoporus balaenopterae* (AB355624)	G	A	T	T	A	G	A	T	T	T	G	A
*Adenocephalus pacificus* (AB548652, AB548653, AB548654)	G	A	G	C	T/C	T	G	A	C	T	T	G/T
*Spirometra decipiens* (KJ599679)	T	G	G	T	G	G	T	T	T	C	T	T
*Sparganum proliferum* (AB015753)	T	G	G	T	G	G	T	T	G	T	T	A

^*^AM712906 is the reference sequence for nucleotide positions.

**Table 3:** The nucleotide patterns used to differentiate diphyllobothriidean tapeworms by the pyrosequencing technique.

Genus species	*D. dendriticum*		*D. ditremum*		*D. latum*		*D. nihonkaiense*		*D. stemmacephalum*		*Di. balaenopterae*		*A. pacificus*		*S. decipiens*		*Sp. proliferum*	
	*nos dif.	positions	nos dif.	positions	nos dif.	positions	nos dif.	positions	nos dif.	positions	nos dif.	positions	nos dif.	positions	nos dif.	positions	nos dif.	positions
*D. dendriticum*		—		—		—		—		—		—		—		—		—
*D. ditremum*	5	A882G, A/C885G, T906C, C912T, T921A		—		—		—		—		—		—		—		—
*D. latum*	4	A882G, A/C885G, C912T, T921A	1	C906T		—		—		—		—		—		—		—
*D. nihonkaiense*	2	A/C885G, T921G	4	G882A, C906T, T912C, A921G	3	G882A, T912C, A921G		—		—		—		—		—		—
*D. stemmacephalum*	3	C912T, T918G, T921A	4	G882A, G885A, C906T, T918G	3	G882A, G885A, T918G	4	G885A, C912T, T918G, G921A		—		—		—		—		—
*Di. balaenopterae*	6	A882G, A897G, G903A, C912T, T918G, T921A	5	G885A, A897G, G903A, C906T, T918G	4	G885A, A897G, G903A, T918G	7	A882G, G885A, A897G, G903A, C912T, T918G, G921A	3	A882G, A897G, G903A		—		—		—		
*A. pacificus*	8	A882G, T888G, T889C, A891T/C, A897T, T906A, T909C, C912T	8	G885A, T888G, T889C, A891T/C, A897T, C906A, T909C, A921G/T	8	G885A, T888G, T889C, A891T/C, A897T, T906A, T909C, A921G/T	9	A882G, G885A, T888G, T889C, A891T/C, A897T, T906A, T909C, C912T	9	A882G, T888G, T889C, A891T/C, A897T, T906A, T909C, G918T, A921G/T	9	T888G, T889C, A891T/C, G897T, A903G, T906A, T909C, G918T, A921G/T		—		—		—
*S. decipiens*	6	A882T, A/C885G, T888G, A891G, A897G, G903T	8	G882T, T888G, A891G, A897G, G903T, C906T, T912C, A921T	7	G882T, T888G, A891G, A897G, G903T, T912C, A921T	6	A882T, T888G, A891G, A897G, G903T, G921T	9	A882T, A885G, T888G, A891G, A897G, G903T, T912C, G918T, A921T	8	G882T, A885G, T888G, A891G, A903T, T912C, G918T, A921T	9	G882T, A885G, C889T, T/C891G, T897G, G903T, A906T, C909T, T912C		—		—
*Sp. proliferum*	9	A882T, A/C885G, T888G, A891G, A897G, G903T, T909G, C912T, T921A	7	G882T, T888G, A891G, A897G, G903T, C906T, T909G	6	G882T, T888G, A891G, A897G, G903T, T909G	8	A882T, T888G, A891G, A897G, G903T, T909G, C912T, G921A	8	A882T, A885G, T888G, A891G, A897G, G903T, T909G, G918T	7	G882T, A885G, T888G, A891G, A903T, T909G, G918T	9	G882T, A885G, C889T, T/C891G, T897G, G903T, A906T, C909G, G/T921A	3	T909G, C912T, T921A		—

^*^Nos dif. is the number of nucleotide differences.
